# Supplementary material for: Determination of Multiple Neurotransmitters through LC-MS/MS to Confirm the Therapeutic Effects of Althaea rosea Flower on TTX-Intoxicated Rats
Source: Molecules. 2023 May 18;28(10):4158. doi: 10.3390/molecules28104158 (PMC10223091; doi:10.3390/molecules28104158)
Supplement: Supplementary file 1 [file molecules-28-04158-s001.zip › molecules-2297298-supplementary.pdf]

Supporting information 1

Tables Captions

Table S1

Summary of intra- and inter-batch precision and repeatability data in rat brain homogenate.

Table S2

The results of matrix effect and relative recovery experiment (n = 6).

Table S3

Stability of the nine analytes in rat brain (n = 3).

Table S1

Summary of intra- and inter-batch precision and repeatability data in rat brain homogenate.

Analytes	Intra-day(n=6) RSD (%)	Inter-day(n=6) RSD (%)	Repeatability (n = 6), RSD (%)
HVA	2.24	3.31	-
5-HIAA	2.65	4.55	4.81
DOPAC	1.67	1.16	-
GABA	1.30	2.40	1.14
5-HT	2.59	1.99	3.59
NE	1.53	1.83	2.44
E	1.65	6.64	4.72
DA	1.57	0.51	5.07
Tyn	3.01	1.27	2.66

Table S2

The results of matrix effect and relative recovery experiment (n = 6).

Analytes	Recovery (%)	RSD (%)	Matrix effect (%)	RSD (%)
HVA	102.96	2.47	103.45	2.34
5-HIAA	102.99	3.31	103.49	3.32
DOPAC	107.53	3.28	108.70	1.77
GABA	101.78	1.16	102.05	0.99
5-HT	94.04	4.05	93.05	4.35
NE	97.97	2.92	97.15	2.97
E	107.59	4.21	105.87	4.22
DA	97.89	2.39	97.86	2.46
Tyn	95.66	4.12	94.94	4.01

Table S3

Stability of the nine analytes in rat brain (n = 3).

Analytes	Freeze & thaw (3 cycles)RSD（%）	4℃(48h) RSD（%）	Room temperature (24h) RSD（%）
HVA	-	-	-
5-HIAA	0.59	0.19	2.84
DOPAC	-	-	-
GABA	0.30	0.76	1.06
5-HT	3.45	1.14	3.68
NE	2.89	0.41	3.68
E	0.89	0.85	3.38
DA	0.25	0.80	3.95
Tyn	0.87	1.29	1.52

Supporting Information 2

Determination of active fractions in ethyl acetate extracted parts of A. rosea flower by LC-QTOF-MS

S1. Experiment

S1.1 Instruments

Agilent-1290 Ultra Performance Liquid Chromatography System, Agilent -6545 High Resolution Quadrupole-Time of Flight Mass Spectrometer (equipped with Dual AJS ESI source, Agilent, USA).

S1.2 Database development

The PubMed database of the National Center for Biotechnology Information (NCBI), the SciFinder database of the American Chemical Society and the CNKI database of Tsinghua University were searched to comprehensively collect and organize the compound data about A. rosea flower in the literature, and combined with the ChemSpider website, Agilent MassHunter Personal The data were collected and collated from the literature, combined with the ChemSpider website, and the Agilent MassHunter Personal Compound Database and Library Manager (PCDL) software to build a database of the chemical composition of *A. rosea* flower, including compound names, structures, chemical formulae and precise molecular weight information.

The data were imported into the mass spectrometry analysis software Mass Hunter for processing, and each compound was identified by searching for the exact molecular weight, mass-to-charge ratio （m/z） of characteristic fragment ions, and isotopic abundance step by step by comparing the compounds with the PCDL database. The molecular ion peaks set for identification were positive ions $[M+H]^+$, $[M+NH_4]^+$, $[M+Na]^+$, and negative ions $[M-H]^-$, $[M+HCOO]^-$, $[M+CH_3COO]^-$.

S1.3 Chromatographic conditions

Chromatographic column: Waters ACQUITY UPLC HSS T3 (3.0×100 mm, 1.8 μm); mobile phase: 0.1% formic acid water（A phase） and acetonitrile（B phase）;

flow rate: 0.3 mL/min; injection volume: 5 μL; column temperature: 40 °C; The gradient elution procedure is shown in Table S4.

Table S4. Gradient elution program.

Time (min)	A%	B%
0	95	5
2	95	5
42	5	95
47.1	95	5
50	95	5

S1.4 Mass spectrometry conditions

Data Dependent Acquisition mode was used to acquire positive/negative ions separately, and Auto-MS/MS mode was used, with scan the mass range from *m/z* 50 to 1500. Mass spectrometry was performed using an electrospray ionization source in positive (ESI+) and negative (ESI-) modes, respectively, with a scan mass range of m/z 50 to 1500.-ESI source conditions were set as follows: (ESI+) voltage 4000 V, (ESI-) voltage 3500 V, atomization gas pressure 45 psi, drying gas flow rate 11 L/min, drying gas temperature 320 °C, sheath gas temperature 350 °C, fragmentation voltage 175 V. The reference ion *m/z* in positive ionization mode was 121.0509 and 922.0098, and the reference ion in negative ionization mode *m/z* was 112.9856 and 1033.9881, respectively. The collision voltages were set at 10 V, 20 V and 30 V for positive ion mode, and -10 V, -20 V and -30 V for negative ion mode in Q-TOF-MS, respectively. The mass axis was calibrated using a mixed standard tuning solution before each sample measurement.

S1.5 Sample pre-treatment

About 250 mg of lyophilized powder of ethyl acetate extract of *A. rosea* flower was accurately weighed and put into a 5 mL centrifuge tube, dissolve it in 5 mL (v/v) of methanol-water (1:1) solution, vortex for 1 min, extract with ultrasound for 30 min, centrifuge at 10000 rpm/min for 5 min. The supernatant was filtered through a 0.22 μm microporous membrane before analysis.

S2. Results

A total of 28 chemical components were detected, including twelve flavonoids, three phenols, three alkaloids, two terpenes, two coumarins, one organic acid and five other types. See the table S5 for specific compound information.

Table S5. Information on chemical composition in ethyl acetate extract of *A. rosea* flower.

No.	RT/min	Compound	Category	molecular mass	Formula	Molecular ion peak （*m*/*z*）	Adduct	Fragment ions(*m*/*z*)
1	17.598	Hematoxylin	Phenols	302.0817	$C_{16}H_{14}O_6$	301.07431	$[M-H]^-$	179.03498,137.02442
2	17.598	Forsythoside A	Phenols	624.20193	$C_{29}H_{36}O_{15}$	623.19413	$[M-H]^-$	461.16645,161.02442
3	7.815	6-Shogaol	Phenols	276.17243	$C_{17}H_{24}O_3$	275.16489	$[M-H]^-$	260.14084,139.11317
4	18.928	(+)-Catechin Hydrate	Flavonoids	308.09145	$C_{15}H_{16}O_7$	309.09852	$[M+H]^+$	291.08480,263.02935
5	13.644	2''-O-Rhamnosylvitexin	Flavonoids	578.16271	$C_{27}H_{30}O_{14}$	577.15601	$[M-H]^-$	457.11295,413.08704,
6	13.644	Buddleoside	Flavonoids	592.17749	$C_{28}H_{32}O_{14}$	637.17541	$[M+HCOO]^-$	283.06204,268.03763
7	17.496	5,7,4’-Trihydroxy-8-methylflavanone	Flavonoids	286.08605	$C_{16}H_{14}O_5$	285.07845	$[M-H]^-$	191.03496,165.01930
8	12.684	quercetin-7-O-β-D-glucopyranoside	Flavonoids	464.09764	$C_{21}H_{20}O_{12}$	465.10484	$[M+H]^+$	303.05045,229.04892
9	12.161	rutin	Flavonoids	610.15445	$C_{27}H_{30}O_{16}$	611.1622	$[M+H]^+$	465.10190,303.04977
10	13.161	astragalin	Flavonoids	448.10285	$C_{21}H_{20}O_{11}$	449.10985	$[M+H]^+$	287.05502,258.05228
11	10.046	isoliquiritin	Flavonoids	418.12905	$C_{21}H_{22}O_9$	463.12575	$[M+HCOO]^-$	257.08157,137.02345
12	18.55	taxifolin	Flavonoids	304.06043	$C_{15}H_{12}O_7$	303.05389	$[M-H]^-$	303.05338,285.04233
13	13.934	naringenin	Flavonoids	272.07066	$C_{15}H_{12}O_5$	273.07483	$[M+H]^+$	227.07121,151.00363
14	17.413	luteolin	Flavonoids	286.04997	$C_{15}H_{10}O_6$	285.0411	$[M-H]^-$	175.07121,151.09781
15	11.524	dihydrokaempferol	Flavonoids	288.06564	$C_{15}H_{12}O_6$	287.0628	$[M-H]^-$	151.01217,135.04345
16	19.473	Retrochalcone	Others	270.09085	$C_{16}H_{14}O_4$	269.0827	$[M-H]^-$	237.05570,133.02951
17	15.11	(-)-e-Viniferin	Others	454.14065	$C_{28}H_{22}O_6$	453.13442	$[M-H]^-$	345.07547,225.05499
18	21.519	1,2-Dihydroxy anthraquinone	Others	240.04449	$C_{14}H_8O_4$	239.03498	$[M-H]^-$	211.04007,167.05024
19	4.303	Phorbol	Others	364.18899	$C_{20}H_{28}O_6$	363.18359	$[M-H]^-$	327.16018,109.02950
20	17.189	Norcimifugin	Others	292.09695	C_{15} $H_{16}O_6$	291.08954	$[M-H]^-$	215.03498,189.05572
21	14.394	Dihydrocapsaicin	Alkaloids	307.21558	$C_{18}H_{29}NO_3$	308.22141	$[M+H]^+$	291.26816,137.06030
22	14.394	Sinapine	Alkaloids	310.16594	$C_{16}H_{24}NO_5$	311.20019	$[M+H]^+$	280.11320,251.06541

No.	RT/min	Compound	Category	molecular mass	Formula	Molecular ion peak （*m/z*）	Adduct	Fragment ions(m/z)
23	7.815	Harmane	Alkaloids	182.08422	$C_{12}H_{10}N_2$	183.09167	$[M+H]^+$	168.0682， 115.05423
24	24.11	Bayogenin	Terpenoids	488.35025	$C_{30}H_{48}O_5$	511.34008	$[M+Na]^+$	475.31780,295.27272
25	32.529	Pseudoginsenoside Rh1	Terpenoids	638.43881	$C_{36}H_{62}O_9$	683.43719	$[M+HCOO]^-$	475.37848,417.33667
26	17.769	Periplogenin	Coumarins	390.24038	$C_{23}H_{34}O_5$	391.24609	$[M+H]^+$	373.28581,355.22522
27	14.837	scopoletin	Coumarins	192.04194	$C_{10}H_8O_4$	191.03498	$[M-H]^-$	176.01151,148.01659
28	10.985	Caffeic acid	Organic acids	180.04109	$C_9H_8O_4$	179.03499	$[M-H]^-$	135.04515,117.03459
